# Discrepancies between subjective importance and actual everyday practice among very old adults and the consequences for autonomy

**DOI:** 10.1007/s00391-021-01981-w

**Published:** 2021-10-08

**Authors:** Luise Geithner, Michael Wagner

**Affiliations:** 1 Cologne Center for Ethics, Rights, Economics, and Social Sciences of Health, Albertus Magnus Platz, 50923 Cologne, Germany; 2 Institute of Sociology und Social Psychology, University of Cologne, Cologne, Germany

**Keywords:** Aged, 80 and over, Long-term care, Multimorbidity, Home nursing, Quality of life, 80 Jahre und älter, Langzeitpflege, Multimorbidität, Häusliche Pflege, Lebensqualität

## Abstract

**Background:**

An individual’s everyday practice in very old age is based on stable dispositions and on the living conditions associated with the person’s stage of life. Age-associated changes in living conditions can cause discrepancies between the person’s dispositions and actual everyday practice that have consequences for the quality of life.

**Objective:**

The aim of this paper is to look more closely at such discrepancies and their associations with living conditions in very old age (long-term care needs, multimorbidity, care tasks) as well as with the feeling of autonomy as an aspect of quality of life.

**Methods:**

A cross-sectional analysis was conducted using data from the first wave of the NRW80+-study on the quality of life of people aged 80 years and over. Data on the subjective importance of five areas of everyday practice and on the frequency of translating these dispositions into practice were used. The analysis focuses on the occurrence of large discrepancies that arise if the subjective importance of an everyday practice deviates considerably from the frequency of performing it.

**Results:**

Among the very old population surveyed, solitary activities are considered the most important, and they are carried out most often. Multimorbidity, long-term care needs, and private care responsibilities are significantly associated with the experience of large discrepancies. Finally, very old adults with large discrepancies report feelings of reduced autonomy.

**Conclusion:**

These initial results show that age-associated changes in living conditions can be accompanied by difficulties for very old adults to put their dispositions into practice. Attention should be paid to such discrepancies to avoid negative consequences for quality of life.

**Supplementary Information:**

The online version of this article (10.1007/s00391-021-01981-w) contains supplementary material, which is available to authorized users.

## Abstract (de)

**Hintergrund:**

Die Alltagspraxis hochaltriger Menschen basiert auf stabilen Dispositionen, aber auch auf den aktuellen, mit der Lebenssituation verbundenen Lebensbedingungen. Mit dem hohen Alter assoziierte Veränderungen der Lebensbedingungen können zu Diskrepanzen zwischen den Dispositionen einer Person und ihrer tatsächlichen Alltagspraxis führen und sich auf die Lebensqualität auswirken.

**Ziel:**

Der Beitrag untersucht diese Diskrepanzen und deren Zusammenhänge mit Lebensbedingungen im hohen Alter (Pflegebedürftigkeit, Multimorbidität, private Pflegetätigkeit) sowie mit Autonomieempfinden als ein Aspekt von Lebensqualität.

**Methoden:**

Es wurde eine Querschnittsanalyse mit Daten der ersten Welle der NRW80+-Studie zur Lebensqualität von Menschen ab 80 Jahren durchgeführt. Es werden Daten zur subjektiven Bedeutung von fünf Bereichen alltäglicher Praxis sowie Angaben zur Häufigkeit der Umsetzung dieser Dispositionen in die Praxis genutzt. Die Analyse fokussiert das Auftreten starker Diskrepanzen, die entstehen, wenn die subjektive Bedeutung der Alltagspraxis von der Häufigkeit ihrer Ausübung deutlich abweicht.

**Ergebnisse:**

In der befragten hochaltrigen Bevölkerung sind solitäre Tätigkeiten subjektiv am wichtigsten; sie werden auch am häufigsten ausgeführt. Multimorbidität, Pflegebedarf und private Pflegetätigkeiten stehen in einem signifikanten Zusammenhang mit dem Auftreten starker Diskrepanzen. Schließlich berichten hochaltrige Menschen mit starken Diskrepanzen ein geringeres Autonomieempfinden.

**Schlussfolgerung:**

Diese ersten Ergebnisse zeigen, dass altersassoziierte Veränderungen der Lebensbedingungen mit Schwierigkeiten für sehr alte Menschen einhergehen können, ihre Dispositionen in Alltagspraxis umzusetzen. Auf solche Diskrepanzen sollte geachtet werden, um negative Auswirkungen auf die Lebensqualität zu vermeiden.

## Introduction and background

Old age is a phase of life that is less institutionally structured. After retirement and the children have left home, older adults are widely relieved of duties imposed by the labor market and family members. This creates an autonomous life situation and the need for people to organize their day to day lives according to their own ideas, attitudes, and orientations. These attitudes and orientations are based on a person’s dispositions that are considered to be relatively stable over the life course and that determine everyday practices, such as daily routines [4, 22]; however, especially for people in advanced old age, health impairments can put new constraints on daily life. Discrepancies between the disposition or proneness to perform certain daily activities and the ability to realize these dispositions might occur. How these discrepancies affect the quality of life in old age, especially the feeling of autonomy, is an open question.

Everyday practice, understood as a behavioral pattern, comprises an interactive normative dimension (way of living) and an expressive esthetic dimension (lifestyle) [1]. This distinction is an analytical one, as both dimensions are an element of the observable everyday practice. Both dimensions are based on a cognitive evaluative dimension that is captured by concepts, such as habitus [4], dispositions or the German expression “Lebensorientierung”. All of these concepts describe an inner orientation, values or attitudes [1, 22]. As conceptualized by the challenges and potentials (CHAPO) model of quality of life of the very old [15, 21], a high correspondence between one’s dispositions and one’s actual everyday practice can be understood as part of a successful life conduct.

Whether dispositions and everyday practice correspond to each other depends on the context of a person’s situation and the living conditions associated with the person’s stage of life [12, 22]. Especially in the last stage of life, the probability of an individual experiencing changes in living conditions with consequences for the daily life is high. Such changes include having to provide care privately for a spouse or having severe health impairments that result in the need for long-term care or multimorbidity [16]. The proportion of older people who report having two or more chronic diseases increases from 66% in the 55–69 years age group to 82% in the 70–85 years age group [24]. Furthermore, the proportion of people being dependent on long-term care according to the German Social Security Code rises sharply in very old age. While 26% of the people in the 80–84 years age group were in need of long-term care in 2019, this proportion rises to 76% in the 90 years and over age group [20]. In Germany, long-term care is still largely provided privately. In 2019, of the people who were cared for in their homes 51% received private care provided by their relatives only [20].

Such changes in people’s living conditions can, however, lead to discrepancies or discontinuities between their dispositions and their actual everyday practice, which may, in turn, result in feelings of dissonance and discomfort [3]. A possible reason for such discrepancies is thought to be the inertia of people’s dispositions. According to Bourdieu [3, 4], a person’s habitus cannot easily adapt to profound changes in living conditions. Bourdieu refers to this as hysteresis. Although a person’s dispositions are constantly adapting to new situations and experiences, the change is never radical as dispositions are based on the conditions of their origin [3, 12]. These assumptions are in line with the central predictions of continuity theory [2] and cognitive dissonance theory [7, 9]. Continuity theory predicts that severe internal or external discontinuities can have negative implications for the older person’s adaptive strategies which might endanger oneʼs identity [2]. In dissonance theory, the notion of cognition includes opinions, beliefs, or attributions. Dissonant relations between relevant cognitions can result in a subjective internal tension [7, 9].

This paper concentrates on the reduction in subjective autonomy as one aspect of individual discomfort. Autonomy “describes the ability to exercise free will, make one’s own decisions, and maintain control over one’s life; over and above external influences” [19]. For example, for an older person, having severe health impairments, moving to a nursing home or having to care for a disabled spouse might make it difficult to continue the daily routines. Activities that are not very important to the person may dominate daily life, for example, when a person is expected to engage in physical activity even though it is not in his or her interests. It may also be that the person can no longer engage in activities that are important, for example, if a sociable person is bound to his or her home due to limited mobility and is not visited very often. Whereas the first can be understood as an overfulfilment of the person’s wishes, the second represents an underfulfilment of the person’s wishes. We assume that both can present challenges in very old age, which may, in turn, elicit feelings of reduced autonomy.

The aim of this paper is to describe the prevalence of the discrepancies between the dispositions and the actual everyday practice of very old people. The paper investigates how these discrepancies vary between individuals based on the extent of their long-term care needs, their degree of multimorbidity and their care responsibilities. We expect discrepancies to be larger for people facing these age-associated developments. Furthermore, it is examined whether the identified discrepancies are associated with a loss of autonomy, which is a central aspect of the quality of life of very old people [15].

## Methods

### Data and sample characteristics

Data from the first wave of the representative survey NRW80+ are employed. The survey on “Quality of Life and Subjective Well-being of the Very Old in North Rhine-Westphalia (NRW80+)” [15, 21] was conducted from August 2017 to February 2018 and included interviews with 1878 people (born before 1 August 1937) living in North Rhine-Westphalia, Germany. The study was funded by the Ministry of Culture and Science of the German State of North Rhine-Westphalia. The interviews were computer-assisted, face-to-face interviews conducted in the home of each target person. The survey has an ethical permit from the Ethics Commission of Cologne University’s Faculty of Medicine.

For the analyses in this paper, a final representative sample of 1863 persons was used, including 176 proxy interviews. For more information on the study design and sample, *see* [8]. All analyses applied sample weighting. The weighted sample contains 1012 persons in the age group 80–84 years, 573 persons in the age group 85–89 years, and 279 persons in the age group 90 years or older; 1187 persons are female, 676 persons are male. Table 1 provides an overview of the sample characteristics regarding level of care, private care responsibilities, and multimorbidity. In level of care 2.7% of the values were missing, in private care 0.3%, and in multimorbidity 1.2%.

**Table 1:** Sample characteristics: level of care, private care responsibilities, and multimorbidity

Categories	Variables	*N*	%
Level of care	No level of care	1210	66.8
	Level 1	58	3.2
	Level 2	214	11.8
	Level 3	193	10.6
	Level 4	95	5.3
	Level 5	43	2.4
Private care responsibilities^a^	Caring for someone privately	112	6.0
	Not caring for someone privately	1746	94.0
Multimorbidity	No diseases	90	4.9
	1 disease	264	14.3
	≥ 2 diseases	1486	80.7

Weighted data

^a^Persons living in nursing homes were coded with 0.

### Measures

#### Dispositions and everyday practice*.*

Dispositions are analyzed by five questions on the subjective importance of five areas of everyday practice (social, physical, solitary, mental, creative). Accordingly, actual everyday practice is measured by five questions on the frequency of translating these dispositions into practice (Table 2; missing values between 0.2% and 2.8%). All questions were answered on a 5-point Likert scale (importance: 1 = not at all important, 2 = less important, 3 = rather important, 4 = very important, 5 = extraordinarily important; frequency: 1 = never, 2 = rarely, 3 = sometimes, 4 = often, 5 = very often).

**Table 2:** Questions on the subjective importance and the frequency of execution of five areas of everyday practice

Questions on the subjective importance	Questions on the frequency of execution	Area
How important is it for you to spend time with others like relatives or friends, to chat or to do something together?	How often do you spend time with others like relatives or friends, chat, or do something with them?	Social
How important is physical exercise for you?	How often do you exercise physically?	Physical
How important is it for you to have peace and time for yourself?	How often do you have peace and time for yourself?	Solitary
How important is it for you to deal with something in greater depth or to study a topic in more detail?	How often do you deal with something in greater depth or study a topic in more detail?	Mental
How important is it for you to be creative or imaginative about something?	How often are you creative or imaginative about something?	Creative

#### Discrepancies*.*

The analyses are based on the assumption that the more important the everyday practice is to a person, the more often he or she wants to carry it out. Both subjective importance and frequency are rated on a 5-point Likert scale. A discrepancy is defined as a deviation between the two ratings. We refer to cases in which the subjective importance of an area of everyday practice is higher than the frequency of execution as a positive discrepancy (importance minus frequency > 0). Cases in which the subjective importance is lower than the frequency of execution are referred to as a negative discrepancy (importance minus frequency < 0); however, only cases in which the deviation between the two ratings is >1 were considered since an exact correspondence between the two scales cannot be assumed. By examining such large discrepancies, we assume that they are subjectively relevant to the person and thus, have greater relevance for the feeling of autonomy.

#### Age-associated living conditions*.*

The level of care is used as one measure of age-associated living conditions. There are five levels of care in Germany, with higher levels reflecting higher needs. The decision about the level of care is based on a professional assessment of the person’s abilities in six different areas: mobility, self-care, cognitive and communicative skills, behavior and psychological well-being, abilities to manage a disease or a therapy, and social contact. The points given in the assessment of these six areas are translated into the five levels of care [14]. This indicator is comparable to the evaluation of autonomy in activities of daily living (ADL) [11, 18]. The level of care and the ADL score are highly correlated in NRW80+ (r = −0.832, *p* < 0.001, *n* = 813); however, the levels of care are based on a more comprehensive assessment using a 5-level scale, which facilitates their interpretation.

Carrying out private care tasks is used as another (dichotomous) measure of age-associated living conditions. In NRW80+, 6.9% of the very old respondents reported that they privately care for another person. Study participants living in nursing homes were not asked this question. They were coded with 0 (= not caring for someone privately).

Furthermore, a multimorbidity score is used to capture the number of diseases for which the respondent was receiving medical treatment at the time of the interview. The questionnaire includes 20 items (1 open category), with each reflecting 1 area of diseases, such as cancer or diabetes. The scale is based on the self-administered comorbidity questionnaire [17], the multimorbidity index in age [6], and the list of diseases used by the German aging survey [23].

#### Autonomy*.*

Autonomy is used as an indicator for quality of life. In NRW80+, it is captured with the question: “Do you shape your life according to your own ideas?” Respondents can provide answers on a 4-point response scale ranging from 1 = not at all to 4 = exactly right.

### Statistical analyses

We used logistic regression to explore significant differences in the odds for large discrepancies associated with age-associated living conditions and linear regression to explore whether there is a significant difference in the feeling of autonomy between the persons experiencing large discrepancies and the persons experiencing no large discrepancies. The occurrence of a large discrepancy is summed up for all five areas of everyday practice. When summing up, all five areas were equally weighed as, to our knowledge, there are no empirical studies that justify different weightings. For the regression analyses, this variable was dichotomized (0 = no large discrepancy, 1 = large discrepancy in one or more areas of everyday practice) to allow for analyses with sufficiently large groups. The analyses were controlled for age and gender as possible confounders. Multicollinearity was ruled out. No systematic pattern of missing values was identified. Results are considered significant if *p* < 0.05. For the logistic regression, Nagelkerke’s pseudo‑R^2^ and the χ^2^-based Omnibus test of model coefficients were used to assess the model fit. For the linear regression, heteroscedasticity was addressed by the use of robust standard errors HC4 [10]. All analyses were carried out with SPSS Version 26.0 (IBM Corp., Armonk, NY, USA).

## Results

### Descriptive overview

**Fig. 1:**
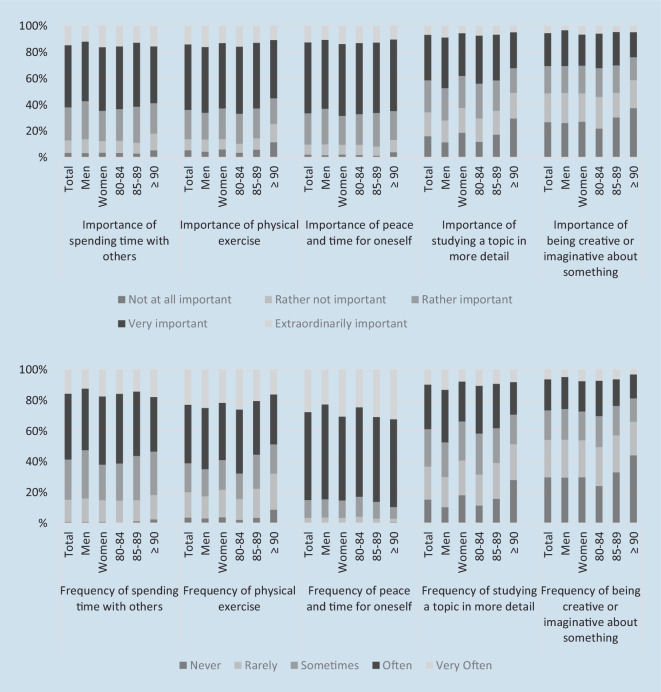
Importance and frequency of the five areas of everyday practice. Weighted data (age groups in years)

First, we give a descriptive overview of the subjective importance and the frequency of corresponding activities in the five areas of everyday practice, including differences by gender and age group (Fig. 1). Of the respondents two thirds (66.4%) rated solitary activities as extraordinarily important or very important, followed by physical exercise (63.9%) and spending time with others (61.9%). For the frequency of performing these daily practices, a similar pattern can be identified. The very old adults surveyed indicated that the area they engage in most frequently is having peace and time for themselves, with 84.9% reporting that they spend time on it very often or often, followed by physical exercise (61.0%) and spending time with others (58.5%).

While 66.0% of the male respondents rated physical exercise as extraordinarily or very important, women assigned the highest importance to peace and time for themselves, with 68.3% reporting that it is extraordinarily or very important. Women reported spending more time with others, while men said they spend more time on physical exercise and studying. In each area of everyday practice, the share of persons assigning extraordinary or high importance is smaller for the older age groups. This is also the case for the frequency of the corresponding everyday practices, except for solitary activities.

### Discrepancies between subjective importance and actual everyday practice

Around 17.3% of the very old population experienced a large positive discrepancy in 1 or more of the 5 areas of everyday practice. Around 15.7% were confronted with a large negative discrepancy. For details see Appendix A of the electronic supplement.

Appendices B1 and B2 show the distribution of large positive and large negative discrepancies for each area of everyday practice, and according to gender, age, and age-associated living conditions. While large positive discrepancies were most common in the areas of social and physical activity, each with around 6% of the very old adults experiencing them, large negative discrepancies were most common for solitary activities with 10.1% of the very old adults experiencing them. Although such discrepancies are not very common in the very old population, Appendix B shows that older people who faced long-term care needs, private care responsibilities, or multimorbidity were affected differently.

### Associations with age-associated living conditions

Table 3 shows the results of the logistic regression analyses for large positive and large negative discrepancies. The explanatory power of the models is significant (*p* < 0.001) with Nagelkerge’s R^2^ = 0.052 for large positive discrepancies and R^2^ = 0.033 for large negative discrepancies. For the experience of a large positive discrepancy in at least one area of everyday practice, significant differences in the odds are found for the three age-associated living conditions. For very old people with higher care levels, very old adults who engage in private care tasks, and very old people with a higher number of diseases, the odds of having a large positive discrepancy are increased. For the experience of a large negative discrepancy in at least one area of everyday practice, significant differences in the odds are found for two of the three age-associated living conditions. The odds of having a large negative discrepancy are increased for very old adults with higher care levels and decreased for very old adults with private care responsibilities.

**Table 3:** Association between age-associated living conditions and the experience of large discrepancies

	One or more large positive discrepancies	One or more large negative discrepancies
	OR (95% CI)	OR (95% CI)
Constant	0.083	0.151
Level of care	1.115* (1.020–1.219)	1.211*** (1.109–1.321)
Caring privately (ref. no)^a^	3.072*** (1.985–4.755)	0.468* (0.222–0.989)
Multimorbidity	1.108*** (1.053–1.166)	0.981 (0.928–1.037)

Weighted data. *N* = 1793. Adjusted for age and gender

*OR* odds ratio, *CI* confidence interval

^a^Persons who do not care for someone privately were defined as reference category.

*p < 0.05, **p < 0.01, ***p < 0.001 (two-sided)

### Associations between discrepancies and the feeling of autonomy

In the last step, a linear regression model (*n* = 1856), adjusted for age and gender, assessed the relationship of experiencing large discrepancies in at least one area of everyday practice with the feeling of autonomy. The analysis shows that for both large positive discrepancies (regression coefficient, *B* = –0.146, 95% confidence interval, CI = –0.259– –0.033, *p* = 0.011) and large negative discrepancies (*B* = –0.246, 95% CI = –0.364– –0.129, *p* < 0.001), the feeling of autonomy decreases significantly.

## Discussion

Our descriptive overview showed that among the very old population of NRW80+, having peace and time for oneself was rated as most important and was most frequently realized in daily life, whereas being creative or imaginative about something was least important and least often realized. For women and men, specific patterns of dispositions and everyday practice could be identified. Among the very old population we studied, social and solitary activities were found to be more important for and more frequently put into practice by women than men, whereas physical exercise and mental activities were found to be more important for and more frequently put into practice by men than women.

In addition, the descriptive overview showed that among people in higher age groups, not only the frequency of the everyday practices was lower, the level of subjective importance of most areas of everyday practice was also lower. A reduced level of aspiration might indicate a coping mechanism. Bourdieu describes the process of individuals who “adjust their aspirations to their objective chances” as “social aging” [4, p. 105]. Whether dispositions change in response to age-associated developments is still an open question. Therefore, further research with longitudinal data or on comparisons with younger age groups would be particularly intriguing. Moreover, it seems that the oldest old compensate for their lower engagement in social, physical, mental, and creative areas of everyday practice by becoming more engaged in solitary activities. Previous studies have found that engaging in solitary activities can improve a person’s well-being if they are performed autonomously [5, 13]; however, further research is needed to understand whether the solitary activity is chosen voluntarily. Our data suggest that this is not always the case. Especially the very old people with long-term care needs have more peace and time for themselves than they wish for.

In the next step, we looked at large positive and large negative discrepancies between the dispositions and the actual everyday practice of the very old people in our sample. The data should be interpreted with caution, as the group of very old people experiencing such large discrepancies was relatively small. Note that due to the different scales used for measuring the importance and the frequency, exact correspondence of the scales cannot be assumed. This is the reason why we examined large discrepancies. In addition, we dichotomized the occurrence of large discrepancies in each area of everyday practice as well as across all five areas to facilitate comparison between people with and without large discrepancies and to allow for regression analyses with sufficiently large groups. Although this approach is limited by a loss of information, further analyses have confirmed the results, which is why we consider them robust. Finally, we cannot draw any conclusions on causal relationships due to our study design.

Across all five areas of everyday practice, the proportion of people who experienced at least one large positive discrepancy was similar to the proportion of people who experienced at least one large negative discrepancy. While the areas of social and physical activities were most often characterized by underfulfilment of the older adults’ wishes, the area of solitary activities was most often characterized by overfulfilment of their wishes.

Next, we identified the age-associated living conditions associated with the odds of experiencing large discrepancies. Higher levels of long-term care needs, suffering from multimorbidity and, being responsible for providing private care increased the odds of experiencing a large positive discrepancy. The odds of experiencing a large negative discrepancy also increased with higher levels of long-term care needs but decreased for people with private care responsibilities. The data show that caring privately for another person seems to foster an underfulfilment of the wish for peace and time for oneself, for social contact, and mental activities which could be due to the fact that private care tasks are often very time consuming. A higher level of long-term care need seems to support both underfulfilment and overfulfilment of certain dispositions. The data show that the former is due to large discrepancies in the areas of physical and creative imaginative activities, while the last is due to large discrepancies in the areas of social and solitary activities. The fact that physical and creative imaginative activities are not carried out to the desired extent is most likely due to the health impairments faced by people with long-term care needs. The finding that both social and solitary activities are realized to a greater extent than desired seems contradictory. One reason could be that very old adults with long-term care needs have social contacts that they did not choose voluntarily (e.g., with the nursing staff or other residents in a nursing home). At the same time, people in need of long-term care may have more time for themselves, for example, due to limited mobility or because they live in a nursing home.

Finally, we found that situations in which the person’s disposition to a certain area of everyday practice was stronger than the actual everyday practice, were associated with lower subjective autonomy. Similarly, autonomy was reduced in situations in which the person’s disposition to a certain area of everyday practice was weaker than the actual everyday practice. These findings are in line with the predictions derived from continuity and cognitive dissonance theories. A low level of subjective autonomy is a kind of internal tension that has been shown to result either from internal or external discontinuities that threaten successful adaptive strategies or from dissonant relations between relevant cognitions.

## Practical recommendations

- Solitary activities receive the highest importance and are most frequently put into practice by the very old population in Germany.
- The experience of large discrepancies between older adults’ dispositions and their actual everyday practice is significantly associated with multimorbidity, long-term care needs and private care responsibilities.
- Large discrepancies are likely to result in reduced feelings of autonomy. Adequate offers of help may enable people to organize their daily lives according to their own values, attitudes, and orientations.

## Supplementary Information

Appendix A: Percentages for the number of large discrepancies across the five areas of everyday practice, Appendix B: Share of very old persons with a large positive or negative discrepancy

## Declarations

All procedures performed in studies involving human participants or on human tissue were in accordance with the ethical standards of the institutional and/or national research committee and with the 1975 Helsinki declaration and its later amendments or comparable ethical standards. Informed consent was obtained from all individual participants included in the study.
