# Supplementary material for: Discrepancies between subjective importance and actual everyday practice among very old adults and the consequences for autonomy
Source: Z Gerontol Geriatr. 2021 Oct 8;54(Suppl 2):101–7. doi: 10.1007/s00391-021-01981-w (PMC8551113; doi:10.1007/s00391-021-01981-w)
Supplement: Supplementary file 1 — Appendix A: Percentages for the number of large discrepancies across the five areas of everyday practice, Appendix B: Share of very old persons with a large positive or negative discrepancy [file 391_2021_1981_MOESM1_ESM.docx]

**Supplementary Material**

**Appendix A:** Percentages for the number of large discrepancies across the five areas of everyday practice

|  | Large positive discrepancy | Large negative discrepancy |
| --- | --- | --- |
|  | **% (n)** | **% (n)** |
| 0 | 82.7 (1,540) | 84.3 (1,569) |
| 1 | 14.0 (261) | 14.3 (265) |
| 2 | 2.7 (51) | 1.4 (25) |
| 3 | 0.5 (3) | 0.1 (2) |
| 4 | 0 (0) | 0 (0) |
| 5 | 0.1 (1) | 0 (0) |

*Weighted data.*

**Appendix B1:** Share of very old persons with a large positive discrepancy

|  | Total | Men | Wo-men |  | 80-84 | 85-89 | ≥ 90 |  | Long-term care need | | | Caring privately | | | Multimorbidity | | |
| --- | --- | --- | --- | --- | --- | --- | --- | --- | --- | --- | --- | --- | --- | --- | --- | --- | --- |
|  |  |  |  | **p value** |  |  |  | **p value** | **No** | **Yes** | **p value** | **No** | **Yes** | **p value** | **No** | **Yes** | **p value** |
|  | **% (n)** | **% (n)** | |  | **% (n)** | | |  | **% (n)** | |  | **% (n)** | |  | **% (n)** | |  |
| Spending time with others | 6.1 (113) | 5.7 (38) | 6.4 (76) | .504 | 5.4 (54) | 6.1 (35) | 8.7 (24) | .122 | 5.7 (69) | 7.0 (43) | .280 | 5.2 (90) | 20.7 (23) | <.001 | 4.3 (15) | 6.6 (98) | .101 |
| Physical exercise | 6.2 (115) | 4.8 (32) | 7.1 (83) | .048 | 4.4 (44) | 8.4 (48) | 8.7 (24) | .001 | 3.9 (47) | 10.5 (64) | <.001 | 6.4 (111) | 3.6 (4) | .280 | 3.7 (13) | 6.8 (100) | .031 |
| Having peace and time for oneself | 2.2 (40) | 1.8 (12) | 2.3 (27) | .450 | 2.9 (29) | 1.6  (9) | 0.7  (2) | .054 | 2.7 (33) | 1.2  (7) | .032 | 1.7 (29) | 8.3 (9) | <.001 | 1.4  (5) | 2.3 (34) | .309 |
| Studying a topic in more detail | 2.1 (38) | 2.7 (18) | 1.7 (20) | .160 | 2.1 (21) | 2.5 (14) | 1.1  (3) | .418 | 1.9 (23) | 2.6 (15) | .389 | 1.9 (32) | 5.5 (6) | .012 | 2.0  (7) | 2.1 (31) | .882 |
| Being creative or imaginative about sth. | 4.9 (89) | 4.2 (28) | 5.3 (61) | .307 | 3.3 (33) | 6.5 (36) | 7.5 (20) | .002 | 3.5 (42) | 6.7 (40) | .002 | 4.7 (81) | 8.0 (9) | .119 | 3.2 (11) | 5.1 (74) | .130 |

*Weighted data. P values are based on Pearson’s chi square tests.*

**Appendix B2:** Share of very old persons with a large negative discrepancy

|  | Total | Men | Wo-men |  | 80-84 | 85-89 | ≥ 90 |  | Long-term care need | | | Caring privately | | | Multimorbidity | | |
| --- | --- | --- | --- | --- | --- | --- | --- | --- | --- | --- | --- | --- | --- | --- | --- | --- | --- |
|  |  |  |  | **p value** |  |  |  | **p value** | **No** | **Yes** | **p value** | **No** | **Yes** | **p value** | **No** | **Yes** | **p value** |
|  | **% (n)** | **% (n)** | |  | **% (n)** | | |  | **% (n)** | |  | **% (n)** | |  | **% (n)** | |  |
| Spending time with others | 3.7 (68) | 2.5 (17) | 4.4 (52) | .041 | 2.9 (29) | 3.5 (20) | 6.9 (19) | .007 | 2.6 (31) | 5.9 (36) | <.001 | 3.9 (68) | 0.9 (1) | .104 | 3.4 (12) | 3.8 (56) | .748 |
| Physical exercise | 2.2 (40) | 2.2 (15) | 2.1 (25) | .886 | 2.2 (22) | 2.8 (16) | 0.7 (2) | .150 | 2.1 (25) | 2.6 (16) | .450 | 2.1 (37) | 2.7 (3) | .680 | 2.0 (7) | 2.2 (32) | .834 |
| Having peace and time for oneself | 10.1 (185) | 9.9 (66) | 10.2 (119) | .794 | 8.5 (85) | 10.9  (61) | 14.4  (39) | .012 | 8.1 (97) | 14.0  (84) | <.001 | 10.7 (183) | 0.9 (1) | .001 | 9.2  (32) | 10.3 (151) | .544 |
| Studying a topic in more detail | 0.5 (10) | 0.6  (4) | 0.4  (5) | .621 | 0.5 (5) | 0.5  (3) | 0.4  (1) | .949 | 0.7 (8) | 0.3 (2) | .381 | 0.5 (9) | 0.9 (1) | .604 | 2.0  (7) | 0.2 (3) | <.001 |
| Being creative or imaginative about sth. | 1.0 (19) | 0.8  (5) | 1.2 (14) | .354 | 1.1 (11) | 0.9 (5) | 1.1 (3) | .918 | 1.4 (17) | 0.3 (2) | .034 | 1.1 (18) | 0.9 (1) | .878 | 0.9 (3) | 1.1 (16) | .703 |

*Weighted data. P values are based on Pearson’s chi square tests.*
